# Optimization of Cocoa Pods Maturity Classification Using Stacking and Voting with Ensemble Learning Methods in RGB and LAB Spaces

**DOI:** 10.3390/jimaging10120327

**Published:** 2024-12-18

**Authors:** Kacoutchy Jean Ayikpa, Abou Bakary Ballo, Diarra Mamadou, Pierre Gouton

**Affiliations:** 1 Laboratoire Imagerie et Vision Artificielle (ImVia), Université de Bourgogne, 21000 Dijon, France; patoudiarra@gmail.com (D.M.); pgouton@u-bourgogne.fr (P.G.); 2 Unité de Recherche et d’Expertise Numérique (UREN), Université Virtuelle de Côte d’Ivoire, Abidjan 28 BP 536, Côte d’Ivoire; 3 Laboratoire de Mécanique et Information (LaMI), Université Felix Houphouët-Boigny, Abidjan 22 BP 801, Côte d’Ivoire; aboubak2005@yahoo.fr

**Keywords:** cocoa pod, ensemble learning, color spaces, GLCM, voting, stacking, machine learning

## Abstract

Determining the maturity of cocoa pods early is not just about guaranteeing harvest quality and optimizing yield. It is also about efficient resource management. Rapid identification of the stage of maturity helps avoid losses linked to a premature or late harvest, improving productivity. Early determination of cocoa pod maturity ensures both the quality and quantity of the harvest, as immature or overripe pods cannot produce premium cocoa beans. Our innovative research harnesses artificial intelligence and computer vision technologies to revolutionize the cocoa industry, offering precise and advanced tools for accurately assessing cocoa pod maturity. Providing an objective and rapid assessment enables farmers to make informed decisions about the optimal time to harvest, helping to maximize the yield of their plantations. Furthermore, by automating this process, these technologies reduce the margins for human error and improve the management of agricultural resources. With this in mind, our study proposes to exploit a computer vision method based on the GLCM (gray level co-occurrence matrix) algorithm to extract the characteristics of images in the RGB (red, green, blue) and LAB (luminance, axis between red and green, axis between yellow and blue) color spaces. This approach allows for in-depth image analysis, which is essential for capturing the nuances of cocoa pod maturity. Next, we apply classification algorithms to identify the best performers. These algorithms are then combined via stacking and voting techniques, allowing our model to be optimized by taking advantage of the strengths of each method, thus guaranteeing more robust and precise results. The results demonstrated that the combination of algorithms produced superior performance, especially in the LAB color space, where voting scored 98.49% and stacking 98.71%. In comparison, in the RGB color space, voting scored 96.59% and stacking 97.06%. These results surpass those generally reported in the literature, showing the increased effectiveness of combined approaches in improving the accuracy of classification models. This highlights the importance of exploring ensemble techniques to maximize performance in complex contexts such as cocoa pod maturity classification.

## 1. Introduction

Determining the maturity of cocoa pods on time is essential to guaranteeing the taste quality and yield of cocoa beans, which is essential to the chocolate industry. Indeed, maturity directly influences the taste, aroma, and color of the beans, which are decisive criteria for the chocolate industry. Various methods exist to assess this maturity, but each has significant limitations.

Based on the pods’ external appearance, the visual approach is highly subjective because it depends on the observer’s experience and expertise. Thus, the same batch of pods could be judged differently depending on the person. Although commonly used, this method does not accurately assess internal maturation [1].

On the other hand, the more technical needle penetration method allows a more objective assessment. Still, it requires specialized equipment and entails additional costs and logistical constraints, especially for small producers. Therefore, it is necessary to develop more reliable, accessible, and economical solutions to optimize pod maturity classification.

Due to these limitations, finding reliable and inexpensive methods to determine cocoa pod maturity is essential. This can help cocoa producers or cooperatives of producers optimize their harvest and improve the quality of their cocoa beans, which can positively impact their profitability and the chocolate industry as a whole.

One of the biggest problems in agriculture is the need for correct or complete information about the crops on the farms, the condition of the plants, or even the progress of the products [2,3]. Digital technologies have brought about the digitalization of agricultural practices, making it possible to monitor the evolution of products through databases. It is more than necessary that technology plays a vital role in the process of crop development. Cocoa is one of the most important crops for most producing countries in terms of exports, and for consuming countries, it is a significant import. The peak harvest period for cocoa is usually once or twice a year and spread over several months, depending on the country. Determining the optimal harvest time for cocoa pods ensures their quality [4].

Reliable information on crop state is crucial to optimizing and maximizing cocoa harvests. Precise knowledge of pod maturity makes determining the optimal harvest time possible. However, some pods do not change color even when they reach maturity due to unpredictable climatic conditions, such as variations in temperature and humidity. This phenomenon complicates the visual assessment of maturity and, therefore, can impact the quality of bean production.

The degree of pod maturation directly influences the quality of the beans; immature pods do not contain enough sugar and compromise optimal fermentation [5]. This fermentation stage is essential for developing aromas and ensuring the quality of the cocoa beans. On the other hand, over-mature pods are often dry, and their beans may already be sprouted or affected by fungal diseases, making them unsuitable for processing.

The appearance of cocoa pods is a vital indicator of maturity, enabling planters to determine the optimal harvest time. To support this identification, artificial intelligence has recently shown its potential in the agricultural field by providing advanced tools for crop analysis [6]. In particular, using color spaces like RGB and LAB allows for more precise extraction, thereby improving the maturity assessment through techniques like the gray level co-occurrence matrix (GLCM). This method provides fine details of pod texture and color, providing quality pre-processing for the following machine learning algorithms to ensure a more reliable classification of pod maturity and optimize harvest.

Our study made the following contributions:

- We preprocessed cocoa pod images by converting RGB color space to LAB space. This transformation improves the differentiation of color and texture levels. The GLCM extractor extracted key features such as texture and contrast from all color channels, enabling an accurate assessment of pod maturity.
- A computer vision solution was developed to analyze features extracted via several classifiers: SVM, XGBoost, KNN, random forest, logistic regression, and decision tree. Majority voting and stacking techniques were applied to combine the three best classifiers, thereby enhancing the robustness and accuracy of the predictions.
- The proposed approach was compared to existing methods, demonstrating significant improvements in terms of accuracy and robustness for classification. Finally, the study highlights this approach’s strengths while identifying areas for improvement in future work.

The manuscript is organized as follows: Section 2 reviews the state of the art on pod maturity and color space-based methods. Section 3 details the materials and methodologies adopted in our approach. Section 4 presents the experiments carried out and the results obtained. Section 5 presents the results of the ablation experiments. Section 6 discusses these results. Finally, Section 7 concludes the study.

## 2. Related Work

The detection of the maturity of agricultural products, particularly that of cocoa pods, has attracted growing interest in image processing research, mainly through color analysis and ensemble learning. These methods provide accurate crop condition assessment, supporting practical applications in agriculture, such as harvest optimization. Several works have been carried out in this area, particularly in the context of research. For example, Lockman et al., in their study, investigated the effectiveness of laser-induced backscatter imaging (LLBI) for determining the firmness and color of cocoa pods at different stages of maturity. Classification of samples according to their maturity resulted in 90% of samples being correctly classified into an unripe group using a laser diode at 658 nm and 95% at 705 nm [7]; Gao et al. proposed a model using ensemble approaches (boosting and bagging) with feature extraction algorithms (LDA and PCA) to predict heart diseases. The experiment results indicated that the bagging ensemble learning method with decision tree feature extraction and PCA performed best [8]. Bueno et al. studied a technique to determine the degree of ripeness of cocoa based on the acoustic sound of cocoa pods. Their approach made it possible to extract recognizable features for the training process and then apply a convolutional neural network to classify the cocoa sound. The experimental model gave an accuracy of 97.46% of the classification system on the maturity of unripe and ripe pods [9]; Galindo et al. investigated an innovative method to determine the maturity of cocoa pods using acoustic sensing technology. Their approach aims to replace the traditional method of hand tapping to identify ripe pods, which relies on the hollow sound produced by less compact beans. A device generates recorded acoustic signals for analysis, and a cepstrum-based technique was used for feature extraction. By applying a model based on the SVM algorithm and using nested cross-validation, they achieved an average test score of 94%, demonstrating the effectiveness of this method in improving harvesting accuracy [10]. Sunday Samuel et al. proposed an ensemble machine learning algorithm, SA-CCT, to accurately predict black rot disease in cocoa trees in southwest Nigeria. By exploiting climate parameters (rainfall and temperature) from the Nigerian Meteorological Agency (NIMET), the model combines a linear algorithm (SARIMA) and a non-linear algorithm (CCT). Evaluation of the model, implemented in Python, yielded high performance, with a precision of 94.29%, a recall of 91.67%, a root mean square error of 0.2357, and an overall accuracy of 94.44%. These results demonstrate the potential of the model to support farmers and extension agents in combating this disease [11]; Ayubi et al. proposed an innovative approach to identify cocoa fruit maturity by combining YOLOv5s with the convolutional block attention module (CBAM). This method was tested on the Cocoa Maturity Dataset TCS 01 and a PT dataset (Cargill, Indonesia). The integration of CBAM aims to improve the feature representation, thereby increasing the detection accuracy. The results show that this approach achieves high accuracy while maintaining a compact model size, making it suitable for practical applications. This method could help improve the productivity of cocoa cultivation [12]. De Oliveira et al. used one approach by studying a deep network-based classification model built on the Inception-Resnetv2 model to identify cocoa pods in their environment and differentiate between ripe cocoa pods before harvest. The results obtained from their study gave an accuracy of 90% [13].

## 3. Materials and Methods

The experiments were conducted using Python programming on a DELL desktop computer in the ImVIA laboratory at Dijon in France. The computer has an Intel(R) Core i7-10700 CPU at 2.90 GHz, 32 GB of RAM, and an NVIDIA Quadro P400 GPU. The models are configured in Python version 3.8.8 using the Keras API version 2.4.3 with the Tensorflow version 2.3 backend.

### 3.1. Dataset

The data used in this study were collected as part of our work to develop solutions to improve cocoa harvests. The CocoaMFD dataset includes images of cocoa pods captured in various stages of maturity, ranging from non-mature to mature. CocoaMFD [14] consists of 1254 images, each containing several pods, thus allowing diversity in visual variations, essential for reliable maturity analysis. The data were collected in the field on a cocoa plantation located in Yakassé 1, Côte d’Ivoire. The images were captured in an uncontrolled environment, reflecting the actual conditions farmers face. This includes natural light, shadow, and weather variations, increasing the dataset’s complexity and realism. The images were taken from different angles and orientations to capture varied details of the texture and color of the pods, two essential characteristics for accurately determining the stage of maturity. By integrating these images into the analysis, CocoaMFD aims to serve as a benchmark for developing computer vision and machine learning algorithms applied to agriculture, allowing researchers and practitioners to study robust approaches for optimizing cocoa harvests.

Preprocessing was carried out, resulting in two classes of images: ripe pods and unripe pods. The description of these classes is presented in the following Table 1:

### 3.2. Color Spaces

Color space is a mathematical model for representing color information as three components that are perceptible, usable, or reproducible by a human or device. Several color models are used in different applications, such as computer graphics, image processing, and computer vision [15]. In this study, we will work in the RGB and LAB color spaces because they showed their effectiveness in our previous study, which focused on a set of spaces [16].

- RGB space: This space is based on the intensity of the red, green, and blue colors [17]. It is used to represent colors intuitively because it directly corresponds to how computer screens and cameras capture colors. The RGB space is sensitive to light variations [18], which can sometimes complicate the analysis of pod maturity under uncontrolled light conditions.
- LAB space: This color space is structured differently, with a brightness component (L) and two color axes (A and B) representing chromatic dimensions: from green to red and from blue to yellow [19]. Unlike RGB, LAB space is designed to be perceptually uniform, meaning it is less sensitive to light variations and color measure [20]. This makes the LAB space particularly effective in differentiating subtle hues, essential for assessing pod maturity.

The combined use of RGB and LAB spaces makes it possible to exploit each of their strengths in the analysis of pod maturity. RGB space captures color information as recorded by devices, making it easier to account for natural shades. On the other hand, the LAB space allows information about brightness and chromatic nuances to be extracted more uniformly and stably, which is advantageous for efficient preprocessing.

By applying both spaces, we obtain a complete analysis of the pods: color variations, which indicate maturity, are amplified in LAB, while variations in light intensity in the RGB space make it possible to refine the detection of pods in natural environments. This complementarity strengthens the accuracy of machine learning models to classify pods into ripe and unripe.

### 3.3. Gray Level Co-Occurrence Matrix (GLCM)

The gray level co-occurrence Matrix (GLCM) is a straightforward texture analysis technique that can easily extract features from images. It quantifies the spatial relationships between pixels [20], capturing how specific gray levels distribute and coexist in an image. This simplicity makes GLCM particularly useful in image recognition and classification applications.

From GLCM, several texture features can be calculated:

- Contrast: Measures the variation in intensity in the image. High contrast indicates marked differences between neighboring pixels.
- Homogeneity: Indicates the proximity of gray levels between neighboring pixels. High homogeneity reflects uniform texture.
- Energy: Represents the intensity distribution and indicates an even texture when the energy is high.
- Entropy: Measures the complexity of the texture. Higher entropy suggests a more disordered and complex texture.

These parameters are defined by the following equations:

(1) $$Contrast=\sum_{i,j=1}^{N}P_{i,j}{\left(i-j\right)}^{2}$$

(2) $$Dissimilarity=\sum_{i,j=1}^{N}P_{i,j}\left|i-j\right|$$

(3) $$Homogeneity=\sum_{i,j=1}^{N}\frac{P_{i,j}}{\left(1+{\left(i+j\right)}^{2}\right)}$$

(4) $$Energy=\;\sqrt{\sum_{i,j=1}^{N}P_{i,j}^{2}}$$

(5) $$Correlation=\sum_{i,j=1}^{N}P_{i,j}\left\lceil \frac{\left(i-\mu_{i}\right)\left(j-\mu_{j}\right)}{\sqrt{\left(\sigma_{i}^{2}\right)\left(\sigma_{j}^{2}\right)}}\right\rceil$$

- $P_{i,j}$: The value in the co-occurrence matrix;
- *i*,*j*: Pixel value indices;
- $\mu_{i},\mu_{j}$: Mean of pixel values *i* and *j*;
- $\sigma_{i},\sigma_{j}$: Variance of pixel values *i* and *j*.

GLCM is widely used in image classification, biomedical analysis, object recognition, and satellite imagery, among other fields. In detecting the maturity of cocoa pods, GLCM captures texture variations between ripe and unripe pods, thus improving the accuracy of machine learning models for image classification.

Algorithm 1 extracts textural features (energy, correlation, dissimilarity, homogeneity, and contrast) based on the gray Level co-occurrence matrix (GLCM) for each color channel (RGB or LAB) and for various distances and angles. The results are stored in a consolidated DataFrame for further analysis.
**Algorithm 1:** GLCM feature extraction for an image datasetFUNCTION GLCM _extractor(dataset)// Initialize the main data frame to store the characteristics     INITIALIZE image_dataset AS EMPTY DataFrame     FOR each image in the dataset Do        // Initialize a data frame to store the characteristics of the current image.          INITIALIZE df AS EMPTY DataFrame         // For each color channel (RGB or LAB)         FOR each channel i IN [0, 1, 2] Do              img = dataset[image, :, :, i]                          // For each distance (1, 3, 5)            FOR each distance d IN [1, 3, 5] Do                 // Calculate GLCM matrix for horizontal direction (angle = 0)                 GLCM = greycomatrix(img, distances = [d], angles = [0])                  // Extract GLCM features for this distance and channel                 df[‘Energy_’ + i + ‘_d’ + d] = greycoprops(GLCM, ‘energy’)[0, 0]                 df[‘Correlation_’ + i + ‘_d’ + d] = greycoprops(GLCM, ‘correlation’)[0, 0]                 df[‘Dissimilarity_’ + i + ‘_d’ + d] = greycoprops(GLCM, ‘dissimilarity’)[0, 0]                 df[‘Homogeneity_’ + i + ‘_d’ + d] = greycoprops(GLCM, ‘homogeneity’)[0, 0]                 df[‘Contrast_’ + i + ‘_d’ + d] = greycoprops(GLCM, ‘contrast’)[0, 0]             // For every angle (π/4, π/2)             FOR each angle a IN [π/4, π/2] Do                 // Calculate the GLCM matrix for distance 1 and current angle                 GLCM = greycomatrix(img, distances = [1], angles = [a])                 // Extract GLCM features for this angle and channel                 df[‘Energy_’ + i + ‘_a’ + a] = greycoprops(GLCM, ‘energy’)[0, 0]                 df[‘Correlation_’ + i + ‘_a’ + a] = greycoprops(GLCM, ‘correlation’)[0, 0]                 df[‘Dissimilarity_’ + i + ‘_a’ + a] = greycoprops(GLCM, ‘dissimilarity’)[0, 0]                 df[‘Homogeneity_’ + i + ‘_a’ + a] = greycoprops(GLCM, ‘homogeneity’)[0, 0]                 df[‘Contrast_’ + i + ‘_a’ + a] = greycoprops(GLCM, ‘contrast’)[0, 0]         // Add the features extracted from the current image to the main DataFrame         image_dataset.ADD(df)             // Return the DataFrame containing all the extracted features     RETURN image_dataset

The GLCM_extractor function is specifically designed to extract textural features from images, focusing on color channel analysis.

The steps described below:

- **Initialization of the main DataFrame:** A main DataFrame is created to store all the features extracted from each image.
- **Image processing:** Each image in the dataset is analyzed by separating its different color channels (for example, R, G, and B channels for RGB space or channels specific to LAB space).
- **Texture feature extraction:** Textural features are extracted using each channel’s gray level co-occurrence matrix (GLCM).
- **Calculation of GLCM matrices:** The function calculates the GLCM for each channel using various combinations of distances and angles.
- **Extracted features:** From the GLCM matrices, the following features are calculated for each distance and angle: energy, correlation, dissimilarity, homogeneity, and contrast
- **Storing results:** The extracted features for each channel and each configuration (distance/angle) are saved in a temporary image-specific DataFrame and then added to the global frame.
- **Final result:** The function delivers a comprehensive view, returning a consolidated DataFrame containing all GLCM features extracted for each image and each channel, marking the culmination of the process.

This method highlights specific extraction for each color channel, making it possible to capture the textural variations specific to each component. This is particularly useful for applications such as image classification of cocoa pods, where subtle differences between channels play a determining role.

### 3.4. Classification Algorithms

We will classify the collected features after extracting features from our cocoa pod images. We used several supervised classification algorithms, which are presented below.

- Logistic regression (LR): This versatile method is widely used because it can model binomial, multinomial, or ordinal variables. Its applicability spans many fields of activity [21], making it a valuable tool in our research.
- K nearest (KNN): This method uses a machine learning discriminant and is not configurable. It uses proximity to make classifications or predictions about the clustering of an individual data point [22].
- Support vector machine (SVM): It is a discriminative classifier in which the classification is based on the decision plans and their boundaries. It is used effectively for classification and regression. SVM uses multiple kernels and can be categorized based on linear, quadratic, cubic, fine Gaussian, and mean Gaussian kernels [23].
- Extreme gradient boosting (XGBoost): A machine learning model excels in sequential ensemble learning and decision trees. Its boosting technique is particularly useful for reducing errors in predictive data analysis [24].
- Random forest (RF): This model combines the concepts of random subspaces and bootstrap aggregating. It performs learning using multiple decision trees trained on slightly different subsets of data [25].
- Decision trees (DT) are a method for data classification that uses a top-down recursive divide-and-conquer approach [26].

### 3.5. Voting Ensemble Algorithms

The classifier voting algorithm is very efficient. It allows voting on the predictions of a set of classifiers. The algorithm capitalizes on weaknesses in one algorithm that may be an advantage for another classifier. Voting combines the predictions from the classifiers into a final prediction output [27,28]. In our study, we will recover the three best classifiers for each experiment, which will be used for voting.

**Figure 1:**
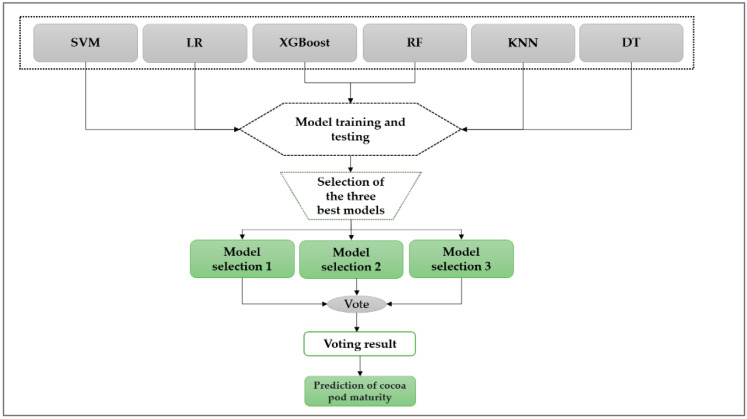
Diagram representing the voting process.

Figure 1 shows the voting process.

This diagram illustrates the voting mechanism used in the study, where predictions from multiple classifiers are aggregated to determine the outcome based on the majority vote.

The voting process illustrated in Figure 1 proceeds as follows:

- Step 1: Test all algorithms used in the study.
- Step 2: Select the three best algorithms from those tested in step 1.
- Step 3: Set up the voting mechanism with the algorithms selected in step 2.
- Step 4: Predict using the voting majority of the selected algorithms.

### 3.6. Set of Stacking Algorithms

**Figure 2:**
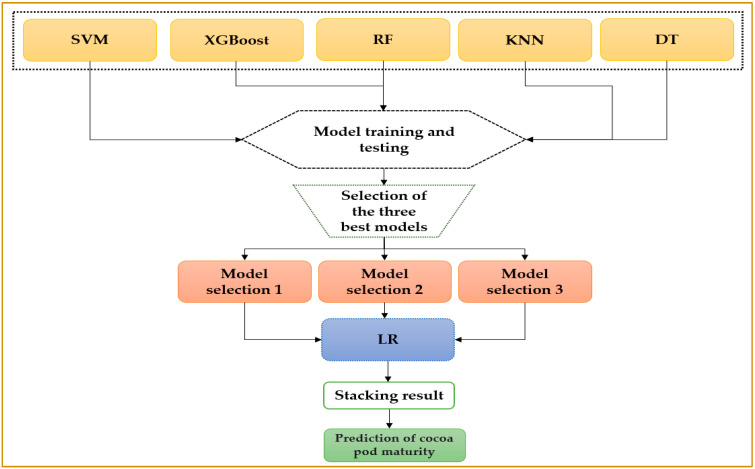
Illustration of the stacking process of the algorithms in our study.

Stacking is a machine learning method that uses meta-learning to integrate multiple algorithms. The method is carried out in two stages. The first stage uses several algorithms to collect their output; the meta-learner merges into another learning algorithm’s stacking structure. In our study, the meta-learner will be the logistic regression algorithm, and the stadium algorithms will be the three best algorithms [29]. Figure 2 illustrates the stacking method used in our study.

This figure depicts the stacking methodology applied in the study, showing how predictions from base models are combined using a meta-model to enhance overall performance and accuracy.

The stacking process is carried out as follows:

- Step 1: All algorithms used in the study, except logistic regression, are tested.
- Step 2: The three best algorithms from step 1 are selected.
- Step 3: The stacking mechanism is set up with the algorithms chosen in step 2.
- Step 4: The meta-learner, logistic regression, is used to optimally combine the predictions from step 3 to obtain an improved prediction.

### 3.7. The General Architecture of Our Methodology

We conducted our study as follows:

- Step 1: Acquisition of our images of cocoa pods for our dataset.
- Step 2: Converting our images into the LAB color space.
- Step 3: We perform feature extraction using GLCM.
- Step 4: We learn our models using the GLCM pair and all of our classifiers. In three steps, for each pair, we recover the three best classifiers for voting and finally carry out the stacking with the three best algorithms using logistic regression, like meta-learner. All this learning will be carried out using data from different color spaces.
- Step 5: We rigorously test our method and evaluate its robustness, ensuring its reliability and applicability.

**Figure 3:**
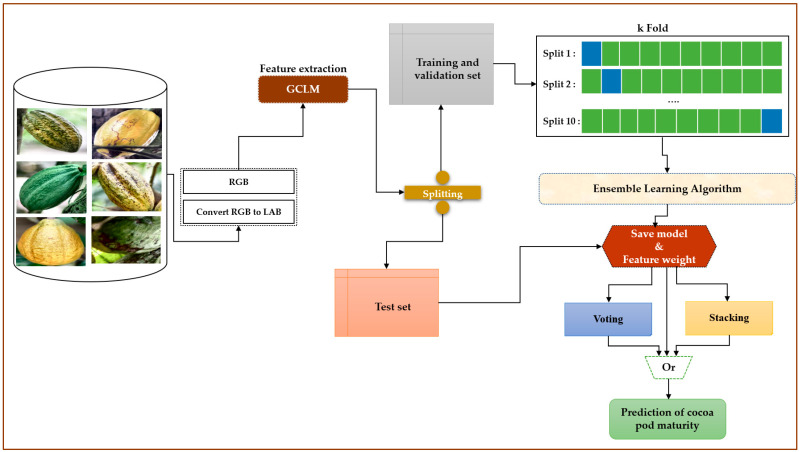
The overall architecture of our method.

Figure 3 illustrates the block diagram of the general methodology adopted.

This figure illustrates the proposed method’s complete workflow, including preprocessing steps, feature extraction, model training, and prediction phases. It highlights key components and their interactions within the system.

### 3.8. Performance Metric

To evaluate the performance of the models studied, we will use various evaluation metrics, including accuracy, precision, mean square error, recall, F1 score, Matthews correlation coefficient (MCC), and the ROC curve. These metrics are calculated using the following formulas. Accuracy, in particular, indicates how well the system has correctly classified data into the appropriate categories.

(6) $$Accuracy=\frac{TP+TN}{TP+FP+TN+FN}$$

Accuracy is the ratio of correctly classified positive images to the total number of genuinely positive images.

(7) $$Precison=\frac{TP}{TP+FP}$$

Recall is the ability of a classifier to determine actual positive results.

(8) $$Recall=\frac{TP}{TP+FN}$$

(9) $$F1\;score=2\times \frac{Precision\times Recall}{Precision+Recall}$$

The F1 score is the weighted average of precision and recall

The Matthews correlation coefficient (MCC) is used in machine learning to measure the quality of classifications.

(10) $$MCC=\frac{TP\times TN-FP\times FN}{\sqrt{\left(TP+FP\right)\left(TP+FN\right)\left(TN+FP\right)\left(TN+FN\right)}}$$

The mean squared error of an estimator measures the average of the squared errors, that is, the mean squared difference between the estimated values and the true value.

(11) $$MSE=\frac{1}{n}\sum_{i=1}^{n}(Yi-\hat{Y}i{)}^{2}$$

These variables are not just terms but the building blocks of understanding the accuracy of a classification model:

- True positives (TP): Images with a true label classified correctly.
- False positives (FP): Images with a false label are classified as positive.
- True Negatives (TN): Images with a false label are classified as negative.
- False Negatives (FN): Images with a true label classified as negative.
- Yi: The actual data in the dataset
- $\hat{Y}i$: The predicted data of the dataset

We used the confusion matrix to evaluate the model’s performance comprehensively and in detail. These tools provide complementary perspectives on analyzing the model’s effectiveness.

The confusion matrix, on the other hand, is a powerful tool that provides a granular view of model performance. It highlights true positives, false positives, and false negatives, which are instrumental in calculating key metrics like precision, recall, and F1 score. More importantly, it helps identify specific model weaknesses, such as a tendency to confuse certain classes, thereby allowing for targeted refinement of the model or training data to improve its robustness.

## 4. Results

The results of our study will be presented for each color space (RGB and LAB), allowing a comparative analysis of the algorithms’ performance according to the characteristics extracted in these different spaces. For each color space, we will detail key performance metrics such as accuracy, precision, recall, F-score, and MCC to highlight the influence of each color space on the effectiveness of classification models.

### 4.1. RGB Color Space

**Figure 4:**
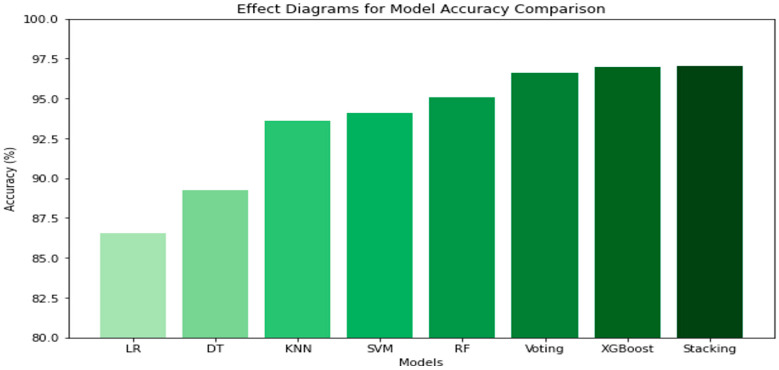
Histogram of model performance comparison (accuracy) in the RGB space.

Table 2 compares the performance of various algorithms using GLCM in RGB to assess cocoa pod maturity. The ensemble models, including stacking and voting, show the best results, with an accuracy of 97.06% for stacking, indicating high precision. Among the other algorithms, XGBoost also stands out with an accuracy of 96.95%. The results confirm that ensemble approaches provide significant improvement in accuracy and robustness. Figure 4 presents the histogram of model performance in the RGB space.

The bars represent the accuracy of each model, with varying colors indicating the performance levels across different algorithms.

**Figure 5:**
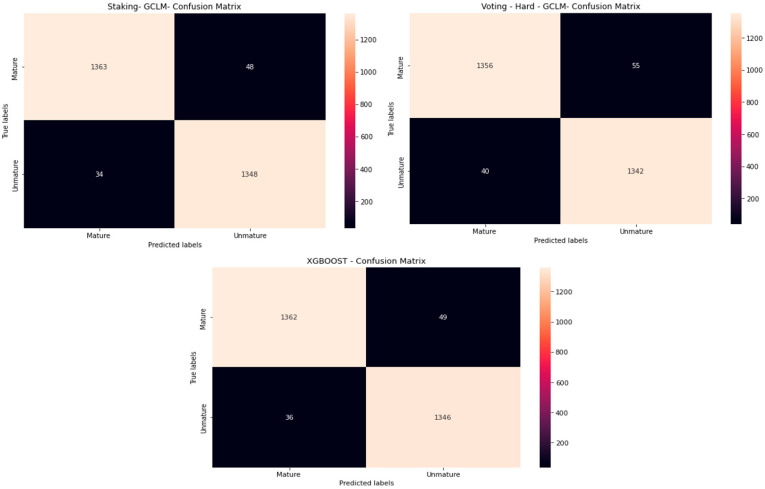
Confusion matrix of the best-performing models in the RGB color space.

Figure 5 presents the confusion matrix of the best-performing algorithms: the stacking and voting ensemble methods and XGBoost, which were selected for their high performance.

This matrix displays the classification results of the best models in the RGB color space, highlighting true positives, false positives, false negatives, and true negatives for each class.

### 4.2. LAB Color Space

**Figure 6:**
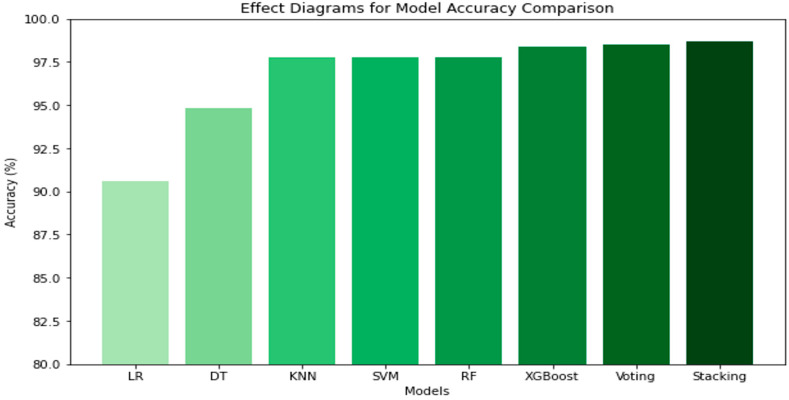
Histogram of model performance comparison (accuracy) in the LAB space.

Table 3 shows the performance of the algorithms in the LAB color space using GLCM for feature extraction. Aggregation techniques, notably voting and stacking, achieve the best scores, with precision, recall, and F-score rates above 98%. Among the individual algorithms, XGBoost also stands out with an accuracy of 98.38%. The high MCC values for the best methods confirm their robustness, and the low MSE for all algorithms shows general classification accuracy. Figure 6 presents the histogram of model performance in the LAB Space.

This diagram presents the accuracy of various machine learning models in the LAB color space. It provides a visual comparison of the results obtained by each model to assess their effectiveness in classifying the data.

**Figure 7:**
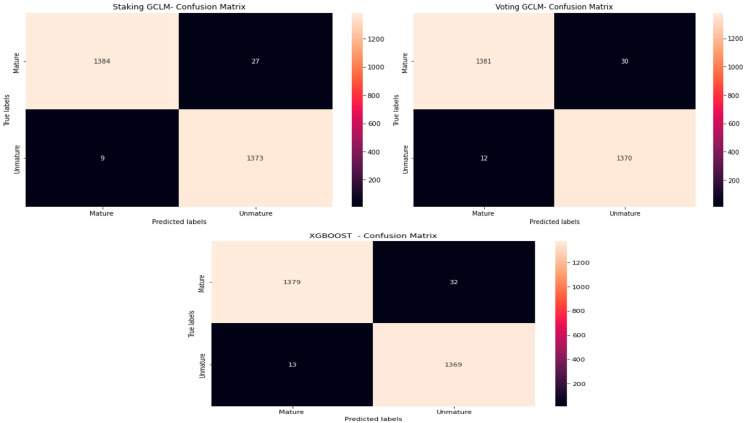
Confusion matrix of the best-performing models in the LAB color space.

Figure 7 shows the confusion matrix of the best algorithms.

This matrix illustrates the classification performance of the top models applied to the LAB color space, showing each class’s distribution of true positives, false positives, false negatives, and true negatives.

### 4.3. Influence of Color Space on Comparison

**Figure 8:**
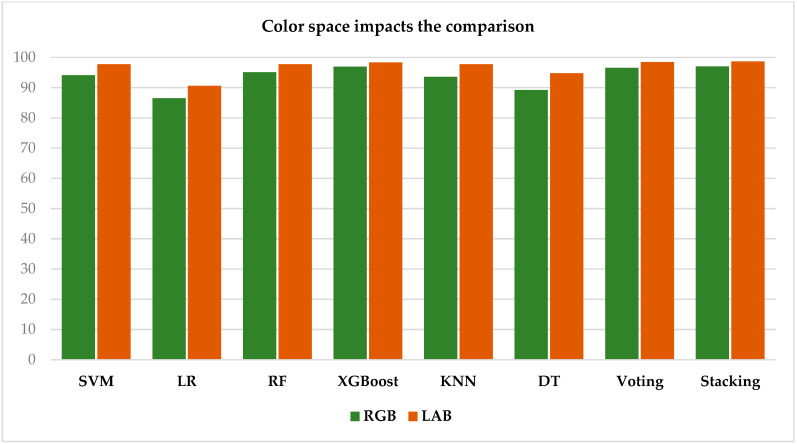
Histogram of algorithm performance in RGB and LAB color spaces.

The performance analysis of the algorithms reveals that the LAB color space consistently yields higher accuracies than the RGB space across all evaluated algorithms. The SVM algorithm achieves an accuracy of 97.78% in the LAB space compared to 94.09% in the RGB space, while the stacking algorithm attains 98.71% in LAB versus 97.06% in RGB. These findings suggest that the LAB color space, with its superior ability to distinguish color nuances, enhances classification precision and boosts algorithm performance in this context. This trend is clearly illustrated in the histogram presented in Figure 8, which summarizes the results for all algorithms.

This histogram visually represents the performance of the evaluated algorithms, highlighting trends and differences in their outcomes for easier comparison and analysis.

### 4.4. State of the Art Comparison

Our study produced better results that exceeded the scores of the methods presented in the literature, as shown in Table 4. This demonstrates the effectiveness of the methodology used in our study. However, some limitations must be taken into account in the different studies.

Oliveira et al. [13] used the Inception-ResNet-V2 model to detect cocoa bean maturity, which was pre-trained on an extensive image database and was successfully used for different recognition tasks image. Using transfer learning with this model can benefit from its performance in solving an image recognition task without needing the same amount of data or calculation time. However, this pre-trained model may not be suitable for the target task, and it may be necessary to adapt it by adjusting some model layers or adding new layers. Additionally, there are often differences between the data used to train the pre-trained model and the target data, which can affect the performance of the adapted model.

Bueno et al. [9] used a technique that involves using audio data from the exocarp (outer part) of the cocoa pod to detect the maturity of cocoa pods. The mel frequency cepstrum was used to extract recognizable features for the ripening process, and a convolutional neural network was used to classify cocoa pods. This process has given satisfactory results, but there are limitations to this technique, such as the quality of the recording influencing the accuracy of the data analysis. If the recording is poor quality, extracting reliable information may be challenging. Additionally, the dataset size (4465 audio files) can be considered a small sample compared to other datasets used in machine learning, which may limit the accuracy of the analysis and generalization of the results obtained from different samples of cocoa beans.

RGB-Voting reached 96.59%, showing a strong performance with the RGB color space. However, this accuracy is lower than that of algorithms using the LAB space, which highlights the importance of the choice of color space in improving classification.

LAB-Voting, which uses the LAB color space, improves the accuracy to 98.49%, outperforming RGB approaches and the performance of Bueno et al.’s study and approaching the best results.

RGB-Stacking shows a performance of 97.06%, which is also relatively high but still lower than that of LAB-Stacking.

LAB-Stacking, with an accuracy of 98.71%, provides the best performance among all compared models, outperforming other approaches in terms of accuracy. This result suggests that using the LAB space and the stacking method allows for a more precise and robust classification.

The results indicate that the LAB approach, combined with ensemble techniques like voting and stacking, generates the best accuracy, showing the importance of choosing color space and ensemble algorithms in classification applications.

## 5. Ablation Experiments

The goal of the ablation experiment is to understand the individual impact of each component on overall performance. We will analyze the effects of RGB and LAB color space, individual algorithms, and ensemble methods (voting/stacking) by comparing the metrics provided.

Table 5 shows that the algorithms’ performance in the LAB space is systematically superior to that of the RGB space. The gains regarding accuracy and MCC metrics are particularly significant, indicating that LAB is more suitable for this problem.

Table 6 shows all algorithms that benefit from using LAB space. Algorithms like KNN, LR, and DT show particularly significant improvements. XGBoost remains efficient in both spaces, but LAB allows for the maximization of its results.

Table 7 demonstrates that ensemble methods consistently outperform individual algorithms regarding results. Using voting and stacking in the LAB space maximizes performance, with stacking providing the best overall accuracy.

The analysis shows that the LAB space systematically outperforms the RGB space for all metrics. The average gain in precision is +3.6% when going from RGB to LAB. Among the algorithms tested, XGBoost, random forest (RF), and KNN stand out as the best performers, particularly in the LAB space. Other algorithms like decision tree (DT) and logistic regression (LR) also significantly improve LAB. Using ensemble techniques, including voting and stacking, helps improve overall results, with optimal performance achieved through stacking. Combining these algorithms exploits the complementarity of the methods, thus achieving better precision. In particular, stacking in LAB space offers the best overall performance with an accuracy of 98.71%, F-score of 98.71%, and MCC of 97.43%. These results show that the joint use of LAB space and ensemble methods is crucial to maximize accuracy.

## 6. Discussion

The results highlight the significant influence of the color space and the classification method on the performance of image analysis algorithms, particularly for models using GLCM (gray level co-occurrence matrix) feature extraction.

Models that use the LAB color space, such as LAB-Voting and LAB-Stacking, consistently outperform those using the RGB color space regarding precision, recall, and F-score. For example, LAB-Stacking achieves an accuracy of 98.71%, while the best RGB model, RGB-Stacking, is limited to 97.06%. This difference shows that the LAB space allows better distinguishing relevant features, improving classification algorithms’ efficiency. This improvement can be attributed to the fact that the LAB space separates the brightness and color components, making it possible to better capture the variations in texture and color of the images of cocoa pods analyzed in the study.

Aggregation techniques, particularly voting and stacking, stand out for their high performance, often outperforming individual models such as SVM, LR, and KNN. For example, LAB-Voting achieves an accuracy of 98.49%, and LAB-Stacking achieves 98.71%, which exceeds the scores obtained by traditional models (SVM and RF) in the same color space. This shows that aggregating multiple models can improve overall accuracy by offsetting the weaknesses of one model with the strengths of another. The stacking method stands out because it uses logistic regression as a meta-learner, thus making it possible to combine the features learned by different base classifiers effectively.

Compared to earlier studies, including those of Oliveira et al. [13] (90.00% accuracy) and Bueno et al. [9] (97.46%), the models presented in this study demonstrate the best effectiveness, particularly the LAB variants of the voting and stacking techniques.

This result indicates that the methodological approach used in this study, including LAB color space and ensemble methods, can significantly advance image classification.

The stacking approach in LAB thus presents a real advance compared to traditional methods, demonstrating increased adaptability and robustness.

## 7. Conclusions

The results of this study show that the choice of the LAB color space, in combination with ensemble methods like voting and stacking, optimizes the accuracy of classification algorithms. Using a meta-learner, the stacking approach shows the ability to improve performance further by capturing complex relationships between predictions from different models.

For continuous improvement, the study could explore optimization techniques and test other meta-learning methods. To assess their robustness in a broader context, the generalization of these models to other types of agricultural images could also be considered. For future work, we will deploy the best solution in a web and mobile application to effectively automate the detection of the maturity of cocoa pods. This solution can also be used to develop other precision agriculture systems based on computer vision.

## Figures and Tables

**Table 1:** Description of the CocoaMFD dataset.

Category	Number of Images	Percentage
Ripe pods	4660	50.1%
Unripe pods	4649	49.9%
Total	9309	100%

**Table 2:** Evaluation of the performance of algorithms in RGB with GLCM.

Algorithms	Accuracy (%)	Precision (%)	MSE	F-Score (%)	Recall (%)	MCC (%)
SVM	94.09	94.09	0.0590	94.09	94.09	88.23
LR	86.53	86.53	0.1346	86.53	86.53	73.21
RF	95.09	95.09	0.0490	95.09	95.09	90.19
XGBoost	96.95	96.95	0.0304	96.95	96.95	93.91
KNN	93.59	93.59	0.0640	93.59	93.59	87.18
DT	89.25	89.25	0.1074	89.25	89.25	78.53
Voting	96.59	96.59	0.0340	96.59	96.59	93.20
Stacking	97.06	97.06	0.02935	97.06	97.06	94.13

**Table 3:** Evaluation of the performance of algorithms in LAB with GLCM.

Algorithms	Accuracy (%)	Precision (%)	MSE	F-Score (%)	Recall (%)	MCC (%)
SVM	97.78	97.78	0.0221	97.78	97.78	95.57
LR	90.61	90.61	0.0938	90.61	90.61	81.24
RF	97.78	97.78	0.0221	97.78	97.78	95.56
XGBoost	98.38	98.38	0.0161	98.38	98.38	96.78
KNN	97.74	97.74	0.0225	97.74	97.74	95.48
DT	94.80	94.80	0.0519	94.80	94.80	89.61
Voting	98.49	98.49	0.0150	98.49	98.49	97.00
Stacking	98.71	98.71	0.0128	98.71	98.71	97.43

**Table 4:** Comparison of the best scores of our methods with methods from the literature.

Models	Accuracy
Oliveira et al. [13]	90.00
Bueno et al. [9]	97.46
RGB-Voting	96.59
LAB-Voting	98.49
RGB-Stacking	97.06
LAB-Stacking	98.71

**Table 5:** Impact of color space.

Color Space	Accuracy (%)	Precision (%)	MSE	F-Score (%)	Recall (%)	MCC (%)
RGB	97.06	97.06	0.0294	97.06	97.06	94.13
LAB	98.71	98.71	0.0128	98.71	98.71	97.43

**Table 6:** Impact of individual algorithms.

Algorithms	RGB (%)	LAB (%)	Gain LAB vs. RGB (%)
SVM	94.09	97.78	+3.69
LR	86.53	90.61	+4.08
RF	95.09	97.78	+2.69
XGBoost	96.95	98.38	+1.43
KNN	93.59	97.74	+4.15
DT	89.25	94.80	+5.55

**Table 7:** Impact of ensemble methods (voting and stacking).

Methods	RGB (%)	LAB (%)	Gain LAB vs. RGB (%)
Voting	96.59	98.49	+1.90
Stacking	97.06	98.71	+1.65

## Data Availability

https://data.mendeley.com/datasets/9msjjh3np6/2 (accessed on 16 December 2024).
